# Pre-Training Effects on Sleep-Dependent Consolidation of Novel Word Learning in Immersive Virtual Reality

**DOI:** 10.3390/jintelligence13110137

**Published:** 2025-10-29

**Authors:** Zhengyu Liu, Lu Jiao

**Affiliations:** 1 School of Humanities and Law, China University of Petroleum (East China), Qingdao 266580, China; liuzhengyu201@mails.ucas.ac.cn; 2 School of Education Science, Qingdao University, Qingdao 266071, China; 3 Brain Cognition and Language Learning Laboratory, Qingdao University, Qingdao 266071, China

**Keywords:** novel word learning, virtual reality, pre-training, consolidation

## Abstract

The present study employed immersive virtual reality (iVR) technology to create a multimodal enriched learning environment and investigated the effects of pre-training on sleep-dependent consolidation of novel word learning. Native Chinese speakers were randomly assigned to either a pre-training group or a control group. Both groups learned two sets of novel words, one on Day 1 and the other on Day 2. All participants completed an explicit recognition task and an implicit semantic priming task on Day 2. The results reveal the sleep-dependent consolidation effects in the implicit measures, with enhanced semantic priming observed for words learned on the previous day following a sleep interval. Moreover, the pre-training yielded additional benefits for sleep-dependent consolidation, as evidenced by the improved sleep-dependent consolidation effects of the pre-training group as compared with the control group. However, no sleep-dependent consolidation and pre-training effects were observed in the explicit recognition task. These findings suggested that pre-training serves as an effective strategy to reduce cognitive load and facilitate novel word learning in iVR environments. This study provides new evidence for the pre-training principle of cognitive load theory from the perspective of sleep-dependent consolidation.

## 1. Introduction

Immersive virtual reality (iVR) presents promising opportunities for foreign language learning by enhancing learner motivation and facilitating semantic integration (Jiao et al. 2025). However, it may also impose challenges by increasing cognitive load (Mayer et al. 2023). A growing body of research on memory consolidation has demonstrated that enriched learning environments can promote sleep-dependent consolidation, such as iVR-based learning contexts (Liu et al. 2024). Nonetheless, from the perspective of Cognitive Load Theory (CLT; Sweller et al. 2019), the realistic scenarios and objects presented in iVR may overwhelm learners’ working memory capacity, thereby impairing memory encoding and subsequent sleep-dependent consolidation (Makransky and Petersen 2021; Mayer et al. 2023). Within the multimedia learning literature, pre-training has been shown to effectively reduce cognitive load by familiarizing learners with the essential features and operations of multimedia environments (Mayer 2021). The present study aims to create an iVR-based learning environment and investigate whether pre-training within this context can enhance sleep-dependent consolidation of newly learned words.

### 1.1. Sleep-Dependent Consolidation Effects on Novel Word Learning

The theoretical basis for sleep-dependent consolidation is grounded in the Complementary Learning System (CLS) model, which posits a dual-phase memory process in word learning (Davis and Gaskell 2009). According to this framework, novel word learning involves a rapid initial familiarization and a slow lexical consolidation (Davis and Gaskell 2009; Kumaran et al. 2016). Sleep-dependent consolidation is closely related to the latter phase, wherein off-line consolidation during sleep facilitates the interaction between newly learned words and the existing mental lexicon. This process enables novel word forms and meanings to become more robustly encoded and to interact dynamically with previously established lexical knowledge (Bakker et al. 2014; Dumay and Gaskell 2007).

Empirical evidence has highlighted the critical role of the learning environment in facilitating sleep-dependent consolidation in novel word learning (Lei et al. 2022; Liu et al. 2024). However, the majority of relevant studies have been confined to traditional foreign language learning contexts, typically involving word-definition or word-image associations (Lei et al. 2022; Liu and van Hell 2020). For instance, Liu and van Hell (2020) employed a word-definition association paradigm in which participants learned one set of novel words on the first day (remote condition with sleep) and another set on the second day (recent condition without sleep). On the second day, a semantic priming task was administered to assess sleep-dependent consolidation effects by comparing the learning performance between remote and recent conditions. The findings revealed enhanced semantic processing for remotely learned novel words, demonstrating the presence of sleep-dependent consolidation. Extending beyond purely verbal learning environments, Lei et al. (2022) investigated a definition-image association paradigm that incorporated nonverbal visual information. Their results indicated superior learning outcomes in the definition-image condition compared to the verbal-only learning condition, providing further support for the advantage of enriched learning environments in sleep-dependent consolidation.

Building on prior research, Liu et al. (2024) employed immersive virtual reality (iVR) technology to simulate a realistic learning environment and investigated its impact on sleep-dependent consolidation of novel word. Adopting a similar experimental design to that of Liu and van Hell (2020), native Chinese participants were assigned to either an iVR-based learning condition or a traditional word–image association condition, and were tasked with learning novel Korean words. Both explicit recognition task and implicit semantic priming task administered on the following day revealed the presence of sleep-dependent consolidation effects across conditions. Notably, in semantic priming task, novel words learned from iVR condition had better sleep-dependent consolidation. These findings suggest that learning in an iVR environment may provide additional benefits for memory consolidation, likely due to the enriched and immersive nature of the learning context.

One potential explanation for the observed link between off-line consolidation and enriched learning environments is that such environments, particularly the iVR environment involving embodied learning, facilitate deeper memory encoding and semantic integration, thereby enhancing the acquisition and consolidation of novel words (Jiao et al. 2024; Li and Jeong 2020; Liu et al. 2024). However, it is important to recognize that the increased cognitive load associated with iVR environments may, in some cases, exceed learners’ working memory capacity. This cognitive overload can reduce the availability of cognitive resources necessary for effective word acquisition and subsequent consolidation (Arztmann et al. 2025; Sweller et al. 2019). As a result, identifying strategies to mitigate cognitive load in iVR-based learning environment is essential to optimize learning outcomes.

### 1.2. Pre-Training Principle in Multimedia Learning

Immersive virtual reality (iVR) is an emerging media for multimedia learning, which is typically implemented through head-mounted displays and handheld controllers to deliver high-resolution visual fidelity and a deeply immersive user experience. In recent years, iVR has gained increasing attention in the field of foreign language education, as it enables learners to engage with virtual objects and environments in interactive, exploratory ways that support experiential learning (Alfadil 2020; Legault et al. 2019).

According to CLT (Sweller et al. 2019), when multimodal inputs exceed an individual’s working memory capacity, cognitive overload occurs, thereby impairing learning performance. CLT distinguishes three types of cognitive load: intrinsic, extraneous, and germane cognitive load (Mayer 2021). Specifically, intrinsic cognitive load is associated with the element interactivity of the learning material (Sweller 2010); extraneous cognitive load arises from the way the learning material is presented; and germane cognitive load refers to the working memory resources invested in managing intrinsic cognitive load rather than extraneous cognitive load (Mayer 2021).

From the perspective of cognitive load, compared with traditional multimedia environments, the educational benefits of iVR may stem from the fact that 360° enrichment increases germane cognitive load by freeing more working memory resources for schema construction. However, one limitation of iVR-based learning lies in the potential for extraneous cognitive overload caused by the perceptual novelty and operational complexity of VR devices (Mayer et al. 2023). Such overload can lead to distraction, overwhelm learners, and consume working memory resources to process elements that do not contribute to knowledge acquisition (Wu et al. 2020).

Pre-training is a well-established instructional strategy designed to reduce extraneous cognitive load (Clarke et al. 2005). Within the multimedia learning literature, the pre-training principle posits that learners can achieve deeper understanding from multimedia materials when they possess relevant prior knowledge (Mayer 2021; Mayer and Fiorella 2021). Furthermore, pre-training may also enhance germane cognitive load by freeing working memory resources from extraneous processing and reallocating them to the management of intrinsic cognitive load, thereby facilitating more effective learning (Mayer 2021). Educational pre-training strategies can generally be categorized into two types: content-oriented pre-training, which involves the delivery of conceptual or domain-specific prior knowledge, and technology-oriented pre-training, which focuses on familiarizing learners with the tools or interfaces used in the learning environment. For iVR environments, extraneous cognitive overload is a factor that have to be taken into consideration (Lawson and Mayer 2025). For instance, Miguel-Alonso et al. (2024) conducted a study in which one experimental group received pre-training that included instruction on the operation of iVR equipment and an overview of the task process. The results indicated that learners in the pre-training group revealed significantly higher levels of engagement and better performance on skill mastery assessments compared to the control group. These findings suggest that technology-oriented pre-training enables learners to manage the demands of the virtual environment more efficiently, thereby supporting improved learning outcomes.

The benefits of pre-training have been widely accepted in educational research (Arztmann et al. 2025; Meyer et al. 2019). Combined with CLT (Sweller et al. 2019), pre-training that targets relevant prior knowledge or procedural skills could alleviate the cognitive demands associated with processing novel information, thereby facilitating more meaningful engagement and interaction with instructional materials (Kester et al. 2004; Mayer and Fiorella 2021). Despite its demonstrated effectiveness in various educational contexts, the role of pre-training in iVR-based foreign language learning remains underexplored. In particular, little empirical attention has been paid on the potential interaction between pre-training and sleep-dependent consolidation, that two factors might influence the acquisition and consolidation of novel words. The present study addresses this gap by examining the effects of pre-training on sleep-dependent consolidation of novel word learning in an iVR environment.

### 1.3. The Present Study

Educational iVR environments are inherently complex due to their enriched sensory inputs and the highly interactive nature of learner-environment interactions (Makransky and Petersen 2021). The pre-training introduced in the present study is designed to reduce the perceptual and operational novelty of the iVR system, decrease extraneous cognitive load, thereby enabling learners to allocate greater cognitive resources to the lexical consolidation of newly learned words. The present study aimed to investigate whether technology-oriented pre-training in a VR context influences sleep-dependent consolidation in novel word learning. Adopting a procedure similar to that of Liu and van Hell (2020), two groups of native Chinese speakers were recruited to learn novel Korean words within an iVR environment over two consecutive days. One group received pre-training focused on iVR device operation, while the control group received basic computer operation training. Following the learning session on Day 2, participants completed a four-alternative forced choice (4AFC) recognition task and a semantic priming task to assess explicit and implicit learning outcomes, respectively (Liu et al. 2024; Palma and Titone 2021).

The present study tested two primary hypotheses. First, drawing on the Complementary Learning Systems (CLS) model (Davis and Gaskell 2009), the off-line consolidation during sleep would facilitate novel words learning (Liu and van Hell 2020). Accordingly, we predicted that words learned on Day 1 (i.e., the remote condition with sleep) would yield superior performance compared to words learned on Day 2 (i.e., the recent condition without sleep). Second, with respect to the role of pre-training, this study examined differences between the pre-training group and the control group. Based on CLT (Sweller et al. 2019), we expected that participants who received VR-based pre-training would learn and consolidate the novel words more effectively, as evidenced by faster response times in 4AFC task and by greater priming effects in the semantic priming task.

## 2. Materials and Methods

### 2.1. Participant

The present study recruited fifty-five native Chinese speakers, and all participants were self-reported right-handed adults. However, data collected from four participants who had high amounts of prior experience with VR equipment were excluded from analyses. In addition, two participants were excluded from data set because they did not complete full experiment. Therefore, the statistical analyses included forty-nine participants, with 23 participants in the pre-training group and 26 participants in the control group. All participants identified Chinese as their first language and reported no prior exposure to or experience with learning Korean. The local ethics committee approved the study and all participants signed written informed consent before formal experiment.

### 2.2. Materials and Procedures

The study included a pre-training session on Day 1, two consecutive learning sessions, and a test session on Day 2. During the pre-training session, the pre-training group received a VR-based operational training, while the control group engaged in basic computer operation. In the learning session, participants learned 15 new words (Set 1) on Day 1 (remote condition), and the other 15 new words (Set 2) on Day 2 (recent condition). In the test session, both groups completed a 4AFC task and a semantic priming task.

#### 2.2.1. VR Pre-Training Session

The pre-training material consisted of a virtual apartment that included a living room, a bedroom, and a bathroom. Participants in the pre-training session were instructed to explore the VR-based operations for 15 min to become familiar with both the immersive environment and the operational features of the VR system (Lawson and Mayer 2025). During the pre-training session, participants performed three types of movements: scanning and navigating within the immersive space; interacting with virtual objects (e.g., picking up and placing objects); selecting target items to hear their corresponding Chinese pronunciations. A researcher monitored the entire session to ensure that participants remained actively engaged with the virtual environment. To maintain engagement throughout the session, participants were prompted to interact with the environment whenever inactivity was detected. It is important to note that, given the study’s focus on the effects of VR technology-oriented pre-training, the virtual environment used during the pre-training session (i.e., a virtual apartment) was intentionally distinct from that used in the subsequent learning session (i.e., a virtual kitchen), in order to isolate the operational familiarity component of pre-training from the content familiarity of learning.

#### 2.2.2. VR Learning Session

Participants in both the pre-training and control groups learned the same set of 30 Korean Hangul characters. The target words were divided into two equal subsets. The first subset (Set 1; remote condition) was learned on Day 1 and tested on Day 2, thereby incorporating an overnight sleep interval. The second subset (Set 2; recent condition) was both learned and tested on Day 2, without a sleep interval. The learning took place in an immersive virtual reality (iVR) environment, utilizing an HTC Vive (HTC Corporation) headset and handheld controllers to provide high-resolution visual fidelity and an immersive interactive experience. Within the virtual kitchen setting, participants were able to physically navigate the environment, visually identify Korean Hangul characters, and hear their pronunciations by pointing a virtual laser at the corresponding 3D objects. The two sets of novel words used in the learning sessions were counterbalanced across participants to control for item-specific effects.

#### 2.2.3. Test Session

In the 4AFC task, each trial began with a 600 ms fixation, followed by the display of a newly learned word along with the four pictures on a computer screen. Participants were instructed to select the correct picture corresponding to the target word. There were 30 trials consisting of 15 trials for remotely learned words and 15 trials for recently learned words. Prior to the formal experiment, participants completed five practice trials to familiarize themselves with the procedure.

**Figure 1:**
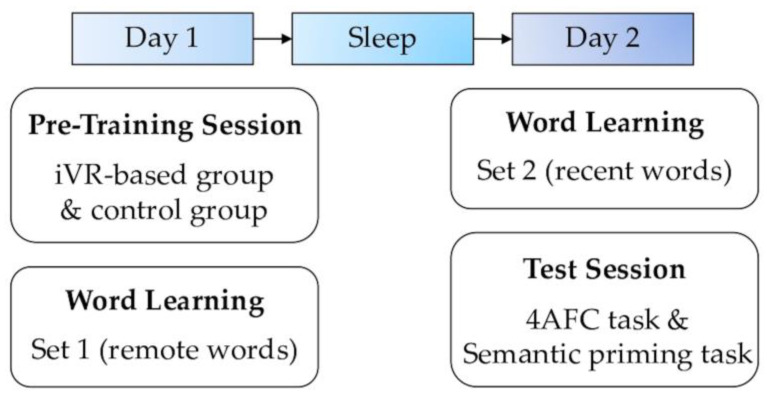
Schematic overview of the tasks.

The semantic priming task involved sleep condition (remote, recent) and relatedness (related/unrelated) variables, resulting in four types of trials (i.e., remote-related, remote-unrelated, recent-related, and recent-unrelated trials). The task comprised a total of 120 trials, with 30 trials allocated to each condition. Each trial began with a fixation for 600 ms, followed by the presentation of a Chinese prime word for 250 ms. After a blank screen for 250 ms, a newly learned word was presented for 1000 ms. Participants were instructed to judge whether the prime word was semantically related to the target word by pressing the left or right button on the keyboard. The response keys were counterbalanced across participants. The practice block consisted of 5 trials to help participants become familiar with the procedure. An overview of the experimental procedure can be seen in Figure 1.

### 2.3. Data Analysis

The response times (RTs) in 4AFC task and semantic priming task were analyzed with mixed-effects models in *R* software (version 4.4.1) using the *lme4* package (Bates et al. 2014). The reason for using mixed-effects models was to achieve the simultaneous consideration of random effects for subjects and items, improving model fit and supporting the generalization of results beyond the specific sample tested. The RTs data that was entered analysis excluded incorrect responses and RTs less than 200 ms or larger than 8000 ms, or beyond M ± 3.0 SD per trial-type. For 4AFC task, the fixed effects included group (pre-training, control), sleep condition (remote, recent), and their interactions. For the semantic priming task, the fixed effects included group (pre-training, control), sleep condition (remote, recent), relatedness (related, unrelated), and their interactions. All variables were sum coded (i.e., control/recent/unrelated = −0.5, pre-training/remote/related = 0.5). Subjects and items were included as random effects. A linear model was conducted for RTs, whereas a logistic model was conducted for accuracy because of the dependent variable’s binominal distribution. Given that the maximal model with maximal random slopes failed to converge, we applied a backward-fitting procedure to select the most comprehensive random-effects structure that would converge (Barr et al. 2013).

## 3. Results

### 3.1. The 4AFC Task

**Figure 2:**
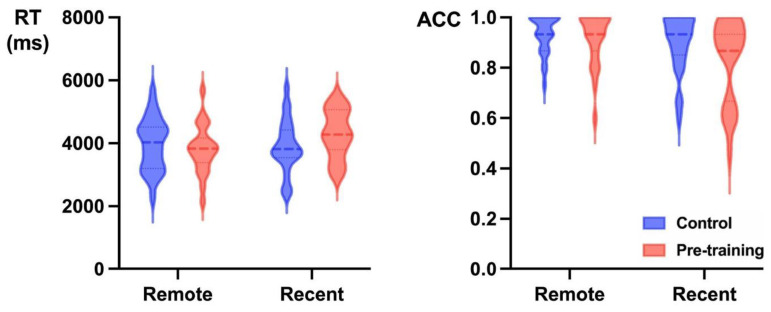
Violin plots for RTs (**left**) and accuracy (**right**) in the 4AFC task. The dotted lines represent the quartiles; the bold lines represent the median.

The RTs for 4AFC task are presented in Figure 2. We fit a linear mixed-effects model with group, sleep condition, and their interactions as fixed effects. It also included the by-subject random slope for sleep condition and the by-item random intercept. There was no significant difference between pre-training group (M = 3935.21 ms, SD = 1743.25) and control group (M = 3835.41 ms, SD = 1794.56; Estimate = 136.99, SE = 178.70, *t* = 0.76, *p* = 0.45, Cohen’s d = 0.09, 95% CI = [0.01, 0.33]), and no difference between remote condition (M = 3805.81 ms, SD = 1704.25) and recent words (M = 3966.32 ms, SD = 1842.14; Estimate = −217.74, SE = 298.87, *t* = −0.73, *p* = 0.47, Cohen’s d = 0.14, 95% CI = [0.01, 0.54]). Moreover, there was no significant interaction between these two variables (Estimate = −573.15, SE = 319.98, *t* = −1.79, *p* = 0.08, Cohen’s d = 0.38, 95% CI = [0.01, 0.81]).

For accuracy of 4AFC task, we fit a logistic mixed-effects model with group, sleep condition, and their interactions as fixed effects. The model included the by-subject random slope for sleep condition and the by-item random intercept. The main effect of group was not significant (Estimate = −0.47, SE = 0.33, *z* = −1.42, *p* = 0.16, Cohen’s d = 0.26, 95% CI = [0.01, 0.62]), with similar performance between pre-training group (M = 88%, SD = 33) and control group (M = 91%, SD = 27). There was no significant difference between remote condition (M = 92%, SD = 26) and recent words (M = 87%, SD = 34), Estimate = 0.61, SE = 0.37, *z* = 1.63, *p* = 0.10, Cohen’s d = 0.33, 95% CI = [0.01, 0.69]. Moreover, the interaction failed to reach significance.

### 3.2. The Semantic Priming Task

**Figure 3:**
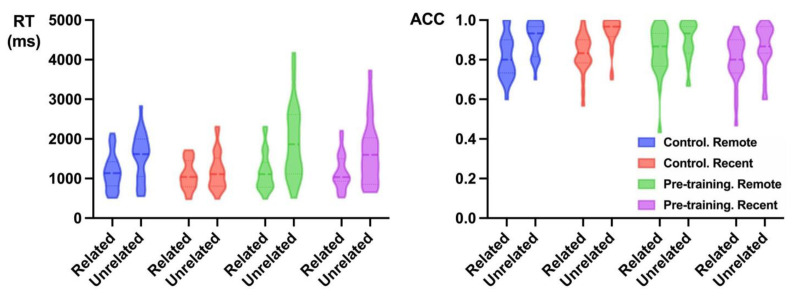
Violin plots for RTs (**left**) and accuracy (**right**) in semantic priming task. The dotted lines represent the quartiles; the bold lines represent the median.

Figure 3 shows the RTs in the semantic priming task for pre-training and control groups. The RTs model for semantic priming task included group, sleep condition, relatedness, and their interactions as the fixed effects, and also included the by-subject random slope for relatedness and the by-item random intercept. The main effect of relatedness was significant (related pairs: M = 1149.62 ms, SD = 1037.19; unrelated pairs: M = 1641.27 ms, SD = 1453.55; Estimate = −474.33, SE = 56.61, *t* = −8.38, *p* < 0.001, Cohen’s d = 0.43, 95% CI = [0.33, 0.53]). Moreover, the relatedness × group interaction was significant (Estimate = −241.29, SE = 113.13, *t* = −2.13, *p* = 0.03, Cohen’s d = 0.22, 95% CI = [0.02, 0.42]), showing an enhanced semantic priming effect (i.e., the differences between related- and unrelated-pairs) in the pre-training group (M_priming effect_ = 622.10 ms) than in the control group (M_priming effect_ = 376.99 ms). And the relatedness × sleep condition interaction also reached significance (Estimate = 202.47, SE = 66.12, *t* = 3.06, *p* = 0.002, Cohen’s d = 0.18, 95% CI = [0.06, 0.30]), showing an enhanced semantic priming effect for remote words with sleep interval (M_priming effect_ = 591.46 ms) than for the recent words (M_priming effect_ = 392.19 ms). There was no other significant effect.

For accuracy, we fit a logistic mixed-effects model with group, sleep condition, relatedness, and their interactions as fixed effects. The model included the by-item random intercept and the by-subject random slope for sleep condition and relatedness. There was a significant main effect of relatedness (related: M = 82%, SD = 38; unrelated: M = 90%, SD = 30; Estimate = −1.00, SE = 0.14, *z* = −6.88, *p* < 0.001, Cohen’s d = 0.55, 95% CI = [0.39, 0.71]). More importantly, the group × sleep condition interaction also reached significance (Estimate = −0.74, SE = 0.24, *z* = −3.11, *p* = 0.002, Cohen’s d = 0.33, 95% CI = [0.07, 0.60]), showing a larger sleep-dependent consolidation effect in the pre-training group (M_sleep effect_ = 4.0%) than in the control group (M_sleep effect_ = −2.4%).

## 4. Discussion

In this study, by utilizing iVR environment, we compared the sleep-dependent consolidation effects of newly learned words between a pre-training group and a control group. Consistent with our predictions, evidence of sleep-dependent consolidation emerged in the form of greater semantic priming effects for words learned in the remote condition (i.e., with sleep) relative to the recent condition (i.e., without sleep). More importantly, technology-oriented pre-training on the iVR device further enhanced sleep-dependent consolidation, as reflected by a larger consolidation effect in the pre-training group compared to the control group. These key findings are discussed in detail in the following subsections.

### 4.1. Sleep-Dependent Consolidation in iVR Environment

Based on the Complementary Learning System (CLS) model (Davis and Gaskell 2009), we expected the remote condition with a sleep interval would facilitate lexical consolidation relative to the recent condition. Although no sleep-dependent consolidation effect was observed in the explicit recognition task (i.e., 4AFC task), a significant interaction between semantic relatedness and sleep condition in semantic priming task provided support for the CLS framework. That is, the sleep interval enhanced the semantic priming effect of remote condition. This finding aligns with previous research conducted in iVR learning environments, where sleep-dependent consolidation has been observed in implicit measures such as semantic priming tasks (Liu et al. 2024). The enhanced semantic integration in the remote condition may be attributed to the role of sleep in offline memory processing. According to the CLS model, novel words learned prior to sleep undergo offline consolidation during the sleep interval, enabling the formation of stronger associations between new lexical items and existing words in the mental lexicon (Tamminen and Gaskell 2013).

Unexpectedly, a significant main effect of relatedness was observed, indicating that novel words learned under both the remote and recent conditions were successfully integrated with existing lexical representations. The efficient integration of recently learned words may be attributed to the multimodal enrichment provided by the iVR learning environment (Jiao et al. 2025; Legault et al. 2019; Li and Jeong 2020). According to embodied cognition theory (Barsalou 2008), immersive learning environments allow learners to physically navigate virtual spaces, interact with meaningful objects, and receive cross-modal input, thereby engaging perceptual and motor systems in the learning process. Moreover, according to cognitive load theory (Sweller et al. 2019), the observed benefit of iVR enrichment for novel word learning may also be attributed to an increase in germane cognitive load (Mayer 2021). The multisensory enrichment afforded by the iVR environment can facilitate schema construction, promote knowledge acquisition, and strengthen the integration of novel words into the existing lexicon. In other words, such enrichment allows learners to allocate more working memory resources to processing intrinsic cognitive load. Consequently, germane cognitive load might increase, reflecting a higher proportion of working memory resources devoted to intrinsic rather than extraneous cognitive load. Consistent with this view, Lei et al. (2022) demonstrated the benefits of incorporating nonverbal information into novel words learning, showing that a definition-image learning environment produced better integration and consolidation of novel words than a verbal-only definition condition.

### 4.2. The Role of Pre-Training

Another key finding of the present study was the effect of pre-training, as evidenced by a significant interaction between group and semantic relatedness in RTs measure, as well as an interaction between group and sleep condition in accuracy measures. In line with the pre-training principle of CLT (Mayer and Fiorella 2021), technology-oriented pre-training can reduce extraneous cognitive load and improve learning performance. Within the context of the iVR environment used in present study, learners were exposed to potentially distracting elements such as complex visual scenes, unfamiliar interface operations, and irrelevant virtual objects. These features may increase extraneous cognitive load because learners must simultaneously process unnecessary interacting elements (Sweller 2010). For learners without prior iVR experience, inappropriate instructional designs may occupy their limited cognitive resources with elements that do not contribute to novel word acquisition. In contrast, participants in the pre-training group had the opportunity to practice operating the equipment and navigating the virtual environment before engaging in the actual learning task. Such prior familiarization with iVR operations may enable more automatic device handling and reduce extraneous cognitive load, thereby facilitating more effective learning (Arztmann et al. 2025; Mayer 2021; Mayer and Fiorella 2021). It is worth noting that both iVR enrichment and pre-training improved learning outcomes, but through different cognitive pathways: iVR enrichment primarily fostered germane cognitive load, whereas pre-training primarily reduced extraneous cognitive load.

Furthermore, according to the Cognitive Affective Model of Immersive Learning (CAMIL; Makransky and Petersen 2021), it is also plausible that the observed improvement in sleep-dependent consolidation was partially driven by individual’s increased intrinsic motivation. Compared to traditional learning environments, the high level of immersion in virtual environments enhances learners’ sense of psychological presence (Makransky and Lilleholt 2018). In the context of the present study, learners who received pre-training experienced lower extraneous cognitive load than those in the control group, which may have resulted in greater enjoyment, higher motivation, and deeper generative processing (Makransky and Petersen 2021). Consistent with prior research, familiarity with the iVR equipment could foster a stronger sense of autonomy and control within the immersive environment. This, in turn, may increase learners’ motivation and engagement, thereby facilitating deeper lexical integration and improved performance on the testing task. While the current findings are consistent with this interpretation, the motivational mechanisms underlying the pre-training effect warrant further empirical investigation.

We acknowledge that certain unexpected findings in the explicit recognition task. While the results demonstrated that iVR-based pre-training facilitated sleep-dependent consolidation in the semantic priming task, this effect did not extend to the explicit recognition (i.e., 4AFC task). One possible explanation is that the accuracy levels for newly learned words were uniformly high across conditions, potentially resulting in a ceiling effect that obscured differences between the pre-training and control groups, as well as between the remote and recent learning conditions. Future research should consider adjusting the difficulty level of explicit recognition tasks to better capture the influence of pre-training interventions and sleep-dependent consolidation processes.

## 5. Conclusions

In the present study, we focus on the iVR learning environment and examine benefits of pre-training on sleep-dependent consolidation of novel word learning. The pre-training effect was examined by comparing learning performance between a pre-training group and a control group, while the sleep-dependent consolidation was assessed by contrasting performance on words learned via remote condition or recent condition. In the implicit semantic priming task, a clear sleep-dependent consolidation effect emerged, with remotely learned words eliciting larger semantic priming effect than recently learned words. More importantly, this consolidation effect was enhanced in the pre-training group, suggesting that VR technology-oriented pre-training facilitated novel word consolidation. These findings provide empirical support for the pre-training principle, revealing that reducing extraneous load through prior familiarization with iVR tools can facilitate sleep-dependent consolidation and improve learning outcomes in immersive environments.

## Data Availability

The raw data supporting the conclusions of this article will be made available by the authors on request.
